# NMR characterisation of the antibiotic resistance-mediating 32mer RNA from the 23S ribosomal RNA

**DOI:** 10.1007/s12104-025-10229-2

**Published:** 2025-04-03

**Authors:** Christina Muhs, Lena Kemper, Christian Richter, Francesca Lavore, Markus Weingarth, Anna Wacker, Harald Schwalbe

**Affiliations:** 1 Center for Biomolecular Magnetic Resonance (BMRZ), Institute for Organic Chemistry and Chemical Biology, Frankfurt am Main, Goethe University, Max-von-Laue-Straße 7, 60438 Frankfurt am Main, Germany; 2 Bijvoet Centre for Biomolecular Research, Department of Chemistry, NMR Spectroscopy, Utrecht University, Padualaan 8, Utrecht, 3584 CH The Netherlands

**Keywords:** m6A, DMA, Erythromycin methyl transferase, Antibiotic resistance, Solution NMR-spectroscopy, 23S ribosomal RNA

## Abstract

The increasing prevalence of antibiotic resistance represents a significant public health concern, underscoring the urgent need for the development of novel therapeutic strategies. The antibiotic effects of macrolides, the second most widely used class of antibiotics, are counteracted by Erm proteins through the methylation of adenosine 2058 of the 23S ribosomal RNA (rRNA) (~ 2900 nucleotides), yielding either monomethylated or dimethylated A2058. This methylation is the molecular basis for preventing macrolides from binding and leads to the development of resistance of bacteria including *Staphylococcus*,* Streptococcus* and *Enterococcus*. While the function of Erm proteins have been thoroughly investigated, the role of the ribosomal RNA in acquiring antibiotic resistance is frequently underestimated, given that the ribosomal RNA is the actual target for methylation. Here, we present the comprehensive ^1^H, ^13^C and ^15^N NMR resonance assignment for the part of the 23S rRNA that serves as the Erm substrate in antimicrobial resistance. Furthermore, we compare the chemical shift signature of the unmethylated to the monomethylated and dimethylated RNA construct and show that changes in the RNA upon methylation are locally restricted. The resonance assignments provide a starting point for investigating and targeting the molecular mechanism of the resistance-conferring Erm proteins.

**Supplementary Information:**

The online version contains supplementary material available at 10.1007/s12104-025-10229-2.

## Biological context

Antibiotic resistance represents a grave threat to human health. In 2019, global death total attributed to antibiotic resistance was 1.27 million with the number of cases of associated death being almost four times higher (Murray et al. 2022). Multi-resistant bacteria, such vancomycin-resistant *Enterococcus* (VRE), methicillin-resistant *Staphylococcus aureus* (MRSA), and bacteria with high affinity to resistances like *Streptococcus pneumoniae* play a major role in these case numbers. The majority of these bacteria are gram positive, with *S. aureus* and *S. pneumoniae* being particularly notable examples. Macrolides, a widely employed class of antibiotics, inhibit bacterial protein biosynthesis by binding to the 23S rRNA of the 50 S ribosomal subunit (Vázquez-Laslop and Mankin 2018). The adenosine residue at position 2058 (numbering from *Escherichia coli*, *E. coli*) plays a pivotal role in this binding event (Vester and Douthwaite 2001; Polacek and Mankin 2005; Rowe et al. 2020). Methylation of adenosine 2058 (A2058) by Erm (erythromycin resistance methyltransferase) proteins, encoded by *erm* genes, results in the so-called MLS (macrolide-lincosamide-streptogramin) resistance (Maravić 2004).

**Fig. 1:**
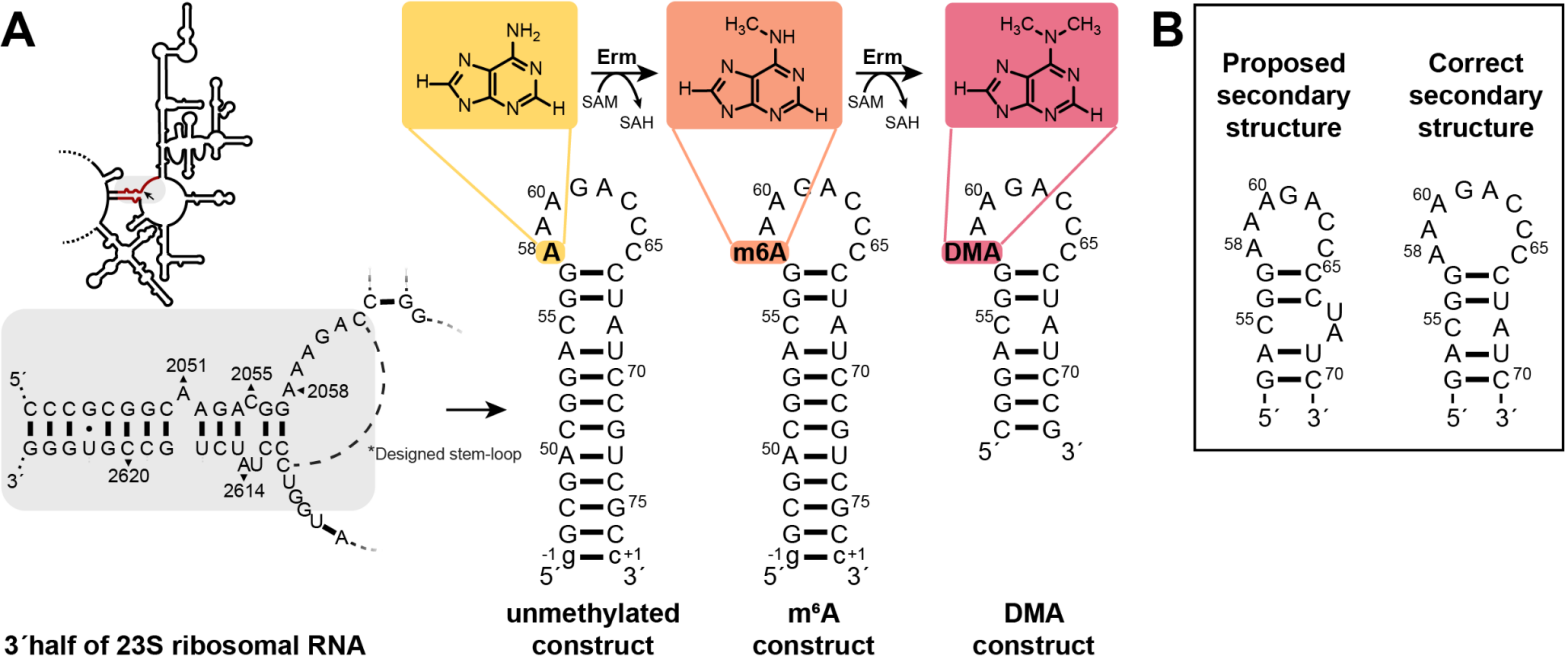
**A**) Schematic overview of the 3´half Domain V of the 23S ribosomal RNA and the relevant area below. In the presence of the co-factor S-adenosyl-methionine (SAM), erythromycin resistance methyltransferase (Erm) transfers a methyl group to the adenosine N6 in a first step (m6A-construct, middle) and with another SAM molecule subsequently in a second step, resulting in the dimethylated RNA (DMA-construct, right). The sequence position in the 23S rRNA Domain V is colored red and covered in grey. The position of A2058 is marked with an arrow. For the unmethylated RNA construct, A2051 was deleted, and the stem-loop was downsized (presented by the dashed lines). The sequence of *S. pneumonia* was used with the numbering of *E.coli*. **B**) Previous studies presented the secondary structure with an apical hepta-loop and C-AU bulge, while our results show a sequence shift resulting in a CA mismatch, GU base pair and an octa-loop

A2058 is located in the unpaired region of the 3´ strand in helix 73 (domain V) of the 23S rRNA (Fig. 1A). N6 of this adenosine is methylated twice by ErmC in a two-step reaction (Denoya and Dubnau 1987). Most of the previous investigations focused on elucidating the molecular mechanism and function of the Erm proteins, while the RNA is an essential part of this reaction and the chemical molecule that is being methylated. Isolated RNA constructs representing the Erm substrate of domain V from *E. coli* and *Bacillus subtilis* (*B. subtilis*) have facilitated investigations of antibiotic resistance occurring through methylation (Vester et al. 1995; Schluckebier et al. 1999). The group of Schluckebier investigated, based on previous design of minimal RNA substrates for 23S rRNA of *E. coli* (Vester et al. 1998), a 32 nucleotide-construct for the 23S rRNA of *B. subtilis*, suitable for structural studies. Although *B. subtilis* is classified only as a facultative pathogen, the relevant RNA sequence is highly conserved in all pathogens with potential for MLS resistance. This highly conserved sequence is predicted to comprise an apical AAAGACC-loop with two GC base pairs followed by a C2055-A2614/U2613 bulge, only the lower stem shows sequence differences between gram negative and gram positive bacteria. Helix and bulge each correspond to the natural sequence, while the loop including the methylation side is the result of truncating helix 73 / domain V. The isolated, unmethylated RNA construct is methylated by Erm (Schluckebier et al. 1999). Deletion of A2051 resulted in a better methylation effect and was therefore used in the following studies (Maravić et al. 2003; Goh et al. 2022).

To facilitate NMR-based structural investigations of the RNA-protein complexes featuring RNAs with different degrees of methylation, we here present the nearly complete ^1^H, ^13^C and ^15^N chemical shift resonance assignment for the unmethylated RNA construct. The unmethylated RNA construct contains 32 nucleotides, comprising a 12-nucleotide long stem with a C-AU bulge and a high GC content, highly conserved in all gram positive pathogens like *Staphylococcus*,* Streptococcus* and *Enterococcus*. The constructed apical hepta-loop consists of the sequence 5´-AAAGACC-3´ and is notably rich in adenosines and cytidines followed by two GC closing base pairs (Fig. 1B).

## Methods and experiments

### Sample-preparation

The unmethylated RNA sample was prepared by in vitro transcription according to a protocol from our group (Wacker et al. 2020) and is described in the following. The methylated (m^6^A-construct) and dimethylated (DMA-construct) RNAs were purchased from Dharmacon. The NMR assignment was based on implementing NMR methodologies as outlined by Ohlenschläger et al. 2003d rtig et al. 2003.

The unmethylated RNA construct with the sequence **5´-gGCGACGGACGGAAAGACCCCUAUCCGUCGCc-3´** was prepared by T7 polymerase-based in vitro transcription from a linearized DNA plasmid template containing a 3’-terminal *Hepatitis Delta* ribozyme (HDV). As T7-Polymerase, the P266L-mutant (Guillerez et al. 2005) was used and prepared as described in He et al. 1997. To maximize transcription efficiency, the terminal base pair was swapped from C/G to g-1/c + 1. A pHDV vector was used to insert the corresponding target sequence derived from annealed primers between the *Eco*RI and *Nco*I restriction sites. The plasmid was transformed into competent *E. coli* DHα-cells, amplified in 2 L of bacterial culture, and isolated and purified using a large-scale DNA isolation kit (Mega Plasmid Kit, Qiagen). Plasmid DNA was linearized using *Hind*III enzyme overnight before performing in vitro transcription.

The transcription mix (20–30 ml) comprised an optimized concentration of 10 mM magnesium acetate and 10 mM rNTPs, together with further 200 mM transcription buffer (Tris/Glutamate, pH 8.1), 2 mM spermidine, 20 mM DTT and 50 ng/ul DNA template. Depending on the labelling scheme ^13^C, ^15^N-labelled nucleotides or unlabeled rNTPs were used. The transcription mix was centrifuged after 7 h at 8500 rpm for 5 min before adding EDTA (0.5 M, pH 8) to a final concentration of 80 mM and 1/10 NaOAc (3 M, pH 5.5), and precipitated with 1 Vol. ice-cold isopropanol at -20 °C overnight. The RNA Pellet was obtained by centrifugation at 8500 rpm for 1 h.

For RNA purification with PAGE (polyacrylamide gel electrophoresis), the RNA pellet was redissolved in 1:1 H_2_O and formamide. Gel electrophoresis was performed at 240 V with water cooling for 2.5 h. The RNA gel bands were visualised with UV-light and cut with a sterile scalpel. Gel pieces were eluted into 0.3 M NaOAc by freezing at -80 °C for 30 min, a subsequent heat shock at 65 °C and shaking at 1300 rpm overnight. The supernatant of the elution mix was filtered through a sterile 0.45 μm cutoff membrane. For the gel granulate, the procedure was repeated once. The supernatants were combined and precipitated with 4 Vol. ethanol for two hours at -80 °C. After centrifugation at 8500 rpm, 0 °C for one hour, RNA pellets were dried and dissolved in water. HPLC was performed with Kromasil RP18 100 A 5 μm 10 × 250 mm column and a gradient from pure 0.1 M triethylammonium acetate (pH 6.1) to 40% with acetonitrile with a flow rate of 2.5 ml/min. HPLC purified RNA was freeze-dried, dissolved in 500 uL H_2_O and precipitated with 2.5 ul 2% LiOCL_4_ (lithium perchlorate) in acetone for 1 h at -80 °C. The precipitated RNA was pelleted by centrifugation at 8500 rpm at 4 °C for one hour. Afterwards the RNA was dissolved in water and folded (5 min at 95 °C) and prepared for NMR measurements. Thereafter, RNA was transferred into NMR buffer (95% H_2_O/5% D_2_O, 25 mM potassium phosphate, 50 mM potassium chloride, adjusted to pH 6.2) using centrifugal concentrators (Vivaspin, 3 kDa molecular weight cut-off). 12% PAA gel electrophoreses (native and denaturing) were performed to confirm the purity and the homogeneous folding of the RNA (SI Fig. 4). The 100% D_2_O sample for the 3D-NOESY-HSQC was prepared by lyophilization of the NMR sample and redissolving in 99.99% D_2_O to keep the buffer salt concentration constant.

The mono methylated (m^6^A) and di methylated (DMA) RNA constructs were obtained from Dharmacon and purified via HPLC as described above. Subsequent sample preparation was conducted as outlined before. The concentrations of the samples were 400 µM for the m^6^A sample and 700 µM for the DMA sample.

### NMR spectroscopy experiments

All NMR measurements were carried out at the BMRZ (Center for Biomolecular Magnetic Resonance) using Bruker spectrometers equipped with following consoles/ probes: 600 MHz (AVIII HD/ 5 mm Prodigy TCI ^1^H,^19^F[^13^C,^15^N]), 700 MHz (AVIII HD/ 5 mm Cryo QCI ^1^H[^13^C,^15^N,^31^P]), 800 MHz (AVIII HD/ 5 mm Cryo TCI ^1^H[^13^C,^15^N]), 800 MHz (AVIII/ 5 mm Cryo TXO ^13^C[^1^H,^15^N]), 1200 MHz (AV NEO/ 3 mm Cryo TCI ^1^H[^13^C,^15^N]). Experiments were performed at 278 K and 298 K (see Table 1), except for temperature series for the experiments 1D^1^H-JR, ^1^H,^15^N-TROSY and ^1^H,^13^C-HSQC temperatures between 278 K and 308 K were measured (SI Fig. 1). NMR spectra were processed using Topspin (versions 4.1.4 and 4.3.0), while the resonance assignment was performed using NMRFAM Sparky 1.470. Regarding calibration of the spectra DSS was used for referencing ^1^H chemical shifts, while ^13^C and ^15^N were indirectly referenced according to the publication of (Wishart et al. 1995).

For the methylated (m^6^A-construct) and dimethylated (DMA-construct) RNA-constructs Table 2 shows the corresponding parameter sets for the NMR measurements.

Spectra were recorded at 278 K and 298 K, after measuring temperature series of 1Ds, TROSY and HSQC spectra (SI Fig. 1).

**Table 1:** List of NMR experiments for the **unmethylated construct** at temperatures 278K^a^ and 298K^b^ at pH 6.2 at BMRZ. Samples are in NMR-buffer in 95% H_2_O/5% D_2_O unless stated otherwise

NMR experiments	Experimental parameters	Characteristic parameters
^1^H,^1^H-NOESY Water suppression: (Hwang and Shaka 1995; Sklenar 1995)	^a^ 600 MHz, ns: 192, sw(f2): 21.70 ppm, sw(f1): 12.00 ppm, aq(f2): 78.6 ms, aq(f1): 31.1 ms, o1(^1^H): 4.7 ppm, o3(^15^N): 153 ppm, relax. delay: 1.0 s, time: 1 d 8 h 22 min ^b^ 900 MHz, ns: 128, sw(f2): 21.05 ppm, sw(f1): 12.25 ppm, aq(f2): 79.0 ms, aq(f1): 44.0 ms, o1(^1^H): 4.698 ppm, o3(^15^N): 117 ppm, relax. delay: 1.0 s, time: 1 d 22 h 54 min	NOE mixing time 250 ms,
^1^H,^15^N-best-TROSY (Solyom et al. 2013)	^a/b^ 600 MHz, ns: 16, sw(f2): 21.04 ppm, sw(f1): 24.66 ppm, aq(f2): 80 ms, aq(f1): 171 ms, o1(^1^H): 4.7 ppm, o2(^13^C): 101 ppm, o3(^15^N): 153 ppm, relax. delay: 0.3 s, time: 1 h 8 min	HN transfer time: 2.7 ms ^1^J_NH_: 10.8 Hz
^1^H,^13^C-HSQC 1 Aromatics 2 C1´-H1´ (Bobenhausen and Ruben 1980)	1 ^b^ 600 MHz, ns: 8, sw(f2): 8.33 ppm, sw(f1): 24.00 ppm, aq(f2): 102 ms, aq(f1): 53 ms, o1(^1^H): 4.7 ppm, o2(^13^C): 143 ppm, relax. delay: 1 s, time: 59 min 2 ^b^ 800 MHz, ns: 4, sw(f2): 8.35 ppm, sw(f1): 12.00 ppm, aq(f2): 77 ms, aq(f1): 80 ms, o1(^1^H): 4.699 ppm, o2(^13^C): 90 ppm, relax. delay: 1 s, time: 29 min	INEPT transfer time 1.25 ms (^1^J_CH_ 200 Hz) C5 decoupling at 98 ppm with 40 ppm bandwidth
^1^H,^13^C-HSQCct All CH, optimized for ribose resonances	^b^ 800 MHz, ns: 4, sw(f2): 8.33 ppm, sw(f1): 100 ppm, aq(f2): 77 ms, aq(f1): 13 ms, o1(^1^H): 4.7 ppm, o2(^13^C): 107 ppm, relax. delay: 1 s, time: 38 min	INEPT transfer time 1.4 ms ^1^J_CH_ 180 Hz
^1^H,^1^H-TOCSY	^b^ 600 MHz, ns: 16, sw(f2): 8.8 ppm, sw(f1): 6.3 ppm, aq(f2): 100 ms, aq(f1): 51 ms, o1(^1^H): 4.7 ppm, o2(^13^C): 101 ppm o3(^15^N): 86 ppm, relax. delay: 1 s, time: 2 h 45 min	C/C-TOCSY mixing time: 30 ms
^1^H,^15^N-cpmgNOESY	^a^ 600 MHz, ns: 256, sw(f2): 21.0 ppm, sw(f1): 103 ppm, aq(f2): 81 ms, aq(f1): 20.5 ms, o1(^1^H): 4.697 ppm, o3(^15^N): 116 ppm, relax. delay: 1 s, time: 23 h	NOE mixing time: 150 ms
^1^H,^15^N-HSQC ^2^J-coupling	^b^ 600 MHz, ns: 64, sw(f2): 10.0 ppm, sw(f1): 85.00 ppm, aq(f2): 85.2 ms, aq(f1): 24.8 ms, o1(^1^H): 4.697 ppm, o2(^15^N): 201.5 ppm, relax. delay: 1 s, time: 5 h 12 min	HN transfer time 16.6 ms ^2^J_HN_ 15 Hz
3D-trhcchco Adenine C2 to C8 (Simon et al. 2001)	^b^ 800 MHz, ns: 16, sw(f3): 8.75 ppm, sw(f2): 22.00 ppm, sw(f1): 58.47 ppm, aq(f3): 73.1 ms, aq(f2): 7.2 ms, aq(f1): 2.7 ms, o1(^1^H): 4.699 ppm, o2(^13^C): 142.5 ppm, o3(^13^C): 142.5 ppm, relax. delay: 1 s, time: 21 h 24 min	CC transfer time 14 ms
^1^H,^13^C-HCCNH Iminos to aromatics	^b^ 700 MHz, ns: 160, sw(f3): 23,0 ppm, sw(f2): 18.00 ppm, sw(f1): 30 ppm, aq(f3): 69.9 ms, aq(f2): 20.2 ms, aq(f1): 0.2 ms, o1(^1^H): 4.7 ppm, o2(^13^C): 139 ppm, o3(^15^N): 154 ppm, relax. delay: 1 s, time: 11 h 30 min	CC TOCSY mixing time 28 ms
3D ^1^H,^13^C-NOESY-HSQC	^b^ 800 MHz, ns: 48, sw(f3): 8.75 ppm, sw(f2): 9.94 ppm, sw(f1): 6.63 ppm, aq(f3): 73.1 ms, aq(f2): 20.0 ms, aq(f1): 20.0 ms, o1(^1^H): 4.7 ppm, o2(^13^C): 137 ppm, relax. delay: 1 s, time: 2 d 16 h 5 min, solvent: 100% D2O	NOE mixing time 200 ms, CH transfer
3D HCCH-TOCSY 1 C1’-C2` 2 C1’-C5` (Kay et al. 1993; Richter et al. 2010)	1 ^b^ 800 MHz, ns: 4, sw(f3): 8.3 ppm, sw(f2): 13.3 ppm, sw(f1): 37.9 ppm, aq(f3): 76.8 ms, aq(f2): 18.0 ms, aq(f1): 8.9 ms, o1(^1^H): 4.7 ppm, o2(^13^C): 91 ppm, relax. delay: 0.9 s, time: 15 h 14 min 2 ^b^ 800 MHz, ns: 4, sw(f3): 8.3 ppm, sw(f2): 13.3 ppm, sw(f1): 37.9 ppm, aq(f3): 76.8 ms, aq(f2): 18.0 ms, aq(f1): 9.9 ms, o1(^1^H): 4.7 ppm, o2(^13^C): 91 ppm, relax. delay: 1 s, time: 18 h 53 min	1 C-C- TOCSY mixing time: 4.7 ms 2 C-C- TOCSY mixing time: 18.8 ms
3D-CNC	^b^ 800 MHz, ns: 16, sw(f3): 24,5 ppm, sw(f2): 34.7 ppm, sw(f1): 12.0 ppm, aq(f3): 67.5 ms, aq(f2): 22.8 ms, aq(f1): 19.9 ms, o1(^13^C): 90 ppm, o3(^15^N): 157 ppm, relax. delay: 0.5 s, time: 1 d 12 h 30 min	CN transfer time ~ 40 ms
3D-HCN	^b^ 700 MHz ns: 16, sw(f3): 9.0 ppm, sw(f2): 71 ppm, sw(f1): 35 ppm, aq(f3): 81.1 ms, aq(f2): 15.3 ms, aq(f1): 25.6 ms, o1(^1^H): 4.7 ppm, o2(^13^C): 114 ppm, o3(^15^N): 158 ppm, relax. delay: 0.95 s, time: 2 d 14 h 33 min	HC-transfer time: 2.8 ms CN-transfer time: 17.5 ms
xf-NOESY (Otting and Wüthrich 1989)	^b^ 600 MHz ns: 64, sw(f2): 20.8 ppm, sw(f1): 11.5 ppm, aq(f2): 70 ms, aq(f1): 27.8 ms, o1(^1^H): 4.7 ppm, o2(^15^N): 145 ppm, o3(^15^N): 201 ppm, relax. delay: 1.2 s, time: 1 d 18 h 10 min	NOE mixing time 200ms

**Table 2:** List of NMR experiments for **m6A/DMA construct** at 1.2 ghz spectrometer at 298 K at BMRZ

NMR experiments	Experimental Parameter	Characteristic parameters
^1^H,^1^H-NOESY Excitations sculpting (Hwang and Shaka 1995)	ns: 128, sw(f2): 20.8 ppm, sw(f1): 14.1 ppm, aq(f2): 164 ms, aq(f1): 49 ms, o1(^1^H): 4.7 ppm, o3(^15^N): 117 ppm, relax. delay: 1.0 s, time: 1 d 18 h 7 min	NOE mixing time 200 ms, JR-delay: 200 µs
^1^H,^1^H-TOCSY (Shaka et al. 1988; Hwang and Shaka 1995)	ns: 32, sw(f2): 8.3 ppm, sw(f1): 6.9 ppm, aq(f2): 102 ms, aq(f1): 61 ms, o1(^1^H): 4.7 ppm, o2(^13^C): 120 ppm o3(^15^N): 100 ppm, relax. delay: 1.5 s, time: 8 h 5 min	C/C-TOCSY mixing time 30 ms
^1^H,^13^C-sfHMQC Aromatics (Schanda et al. 2005)	ns: 512, sw(f2): 9.1 ppm, sw(f1): 24.0 ppm, aq(f2): 71 ms, aq(f1): 18 ms, o1(^1^H): 4.7 ppm, o2(^13^C): 143 ppm relax. delay: 0.5 s, time: 21 h 29 min	HC transfer time: 2.6 ms
^1^H,^13^C-HSQC All CH	^m6A^ ns: 256, sw(f2): 8.33 ppm, sw(f1): 100.00 ppm, aq(f2): 51.2 ms, aq(f1): 4.2 ms, o1(^1^H): 4.7 ppm, o2(^13^C): 107 ppm, relax. delay: 1 s, time: 1 d 17 h 4 min ^DMA^ ns: 400, sw(f2): 8.33 ppm, sw(f1): 100.00 ppm, aq(f2): 51.2 ms, aq(f1): 4.2 ms, o1(^1^H): 4.7 ppm, o2(^13^C): 107 ppm, relax. delay: 1 s, time: 1 d 6 h 22 min	INEPT transfer time 2.8 ms ^1^J_CH_ 180 Hz

### Numbering

For the sake of simplicity, the nucleotide annotation used in the assignment will correspond to the annotation of (#_*E. coli*_ – 2000). For instance, the annotation A58 is used in place of A2058.

Chemical shift perturbations (CSP) were determined by the Euclidean distances d via following equation:

$$\:d=\:\sqrt{\frac{1}{2}({\delta\:}_{H}^{2}+\alpha\:*{\delta\:}_{C}^{2})}$$

δ denotes the chemical shift change of ^1^H and ^13^C in ppm, α is a weighting factor (α = 0.25) for ^13^C based on the gyromagnetic ratio (Getz et al. 2007).

## Extent of assignments and data deposition

### Imino-assignment of the unmethylated RNA reveals the correct secondary structure

The resonance assignment for the unmethylated RNA construct was conducted using a uniformly ^13^C, ^15^N-labelled, a selectively ^13^C, ^15^N-A-labelled, a selectively ^13^C, ^15^N-A/U-labelled, and an unlabelled RNA sample and is described in the following. The NMR experiments used for the assignment of the unmethylated RNA construct are listed in Table 1.

**Fig. 2:**
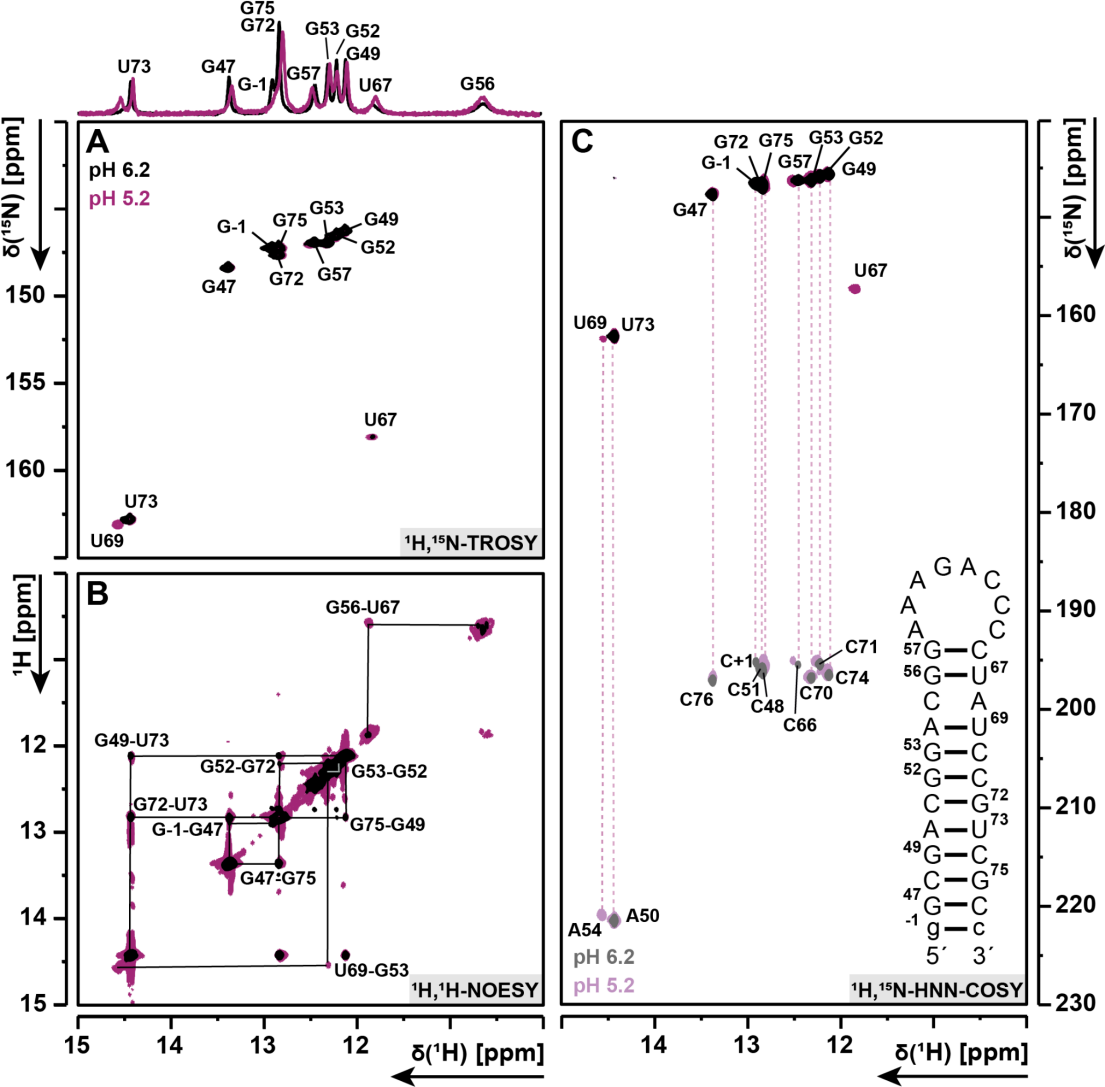
Imino region assignment using **(A)**^1^H,^15^N-TROSY, **(B)**^1^H,^1^H-NOESY, **(C)**^1^H,^15^N-HNN-COSY for imino-proton correlation of the unmethylated RNA construct (right) at pH 6.2 (black) and pH 5.2 (pink) to obtain all imino proton signals at 278 K. Imino proton correlations in **B** are illustrated with the black lines for pH 5.2 (pink). Negative contours in **C** are colored for pH 6.2 grey and for pH 5.2 light pink. Experimental information can be taken from Table 1

The imino proton resonances were initially assigned using ^1^H,^1^H-NOESY (Fig. 2B) and ^1^H,^15^N-TROSY (Fig. 2A) spectra. Base pairing was confirmed by ^1^H,^15^NHNNCOSY (Fig. 2C) spectra. Almost all imino protons were assigned based on sequential NOE contacts at 278 K (pH 6.2), except for G56, U67 and U69. The signals observed at 10.65 and 11.84 ppm were assigned to the wobble base pair G56-U67. The imino proton signal for U69, located adjacent to the C55-A68 mismatch, was not observed at 278 K (pH 6.2). Furthermore, we observed an isolated guanosine imino proton signal, which was assigned as the loop-closing G57, implying an octa-loop instead of the hepta-loop (Fig. 1B). The imino proton exchange rate is known to decrease at moderately lower pH values (Nonin et al. 1997; Snoussi and Leroy 2001). In order to confirm the tentative assignments and observe all imino proton resonances anticipated for our revised secondary structure model, the pH was adjusted from pH 6.2 to 5.2.

Consequently, at pH 5.2, we were able to detect the NOE signals between G56 and U67 missing at pH 6.2, and also detected the previously missing imino proton signal of U69 at 14.55 ppm, consistent with canonical base pairing to A54 (Fig. 2C). In summary, 100% of the imino resonances were assigned. With regard to the CA mismatch, no imino signal for either a protonated adenosine or a protonated cytidine was observed. Moreover, all aromatic H6 resonances for the cytidines, except for C63-C66 (loop signals) and C55 were assigned using a ^1^H,^15^N-CPMG-NOESY combined with the assignment of corresponding H42 and H41 amino proton resonances from the ^1^H,^1^H-NOESY. Only the H2 resonances of A50 and A54 could be assigned by their NOE contacts to their base pairing uridines, as the other five adenosines are not involved in base pairs.

### Aromatic and ribose assignment

**Fig. 3:**
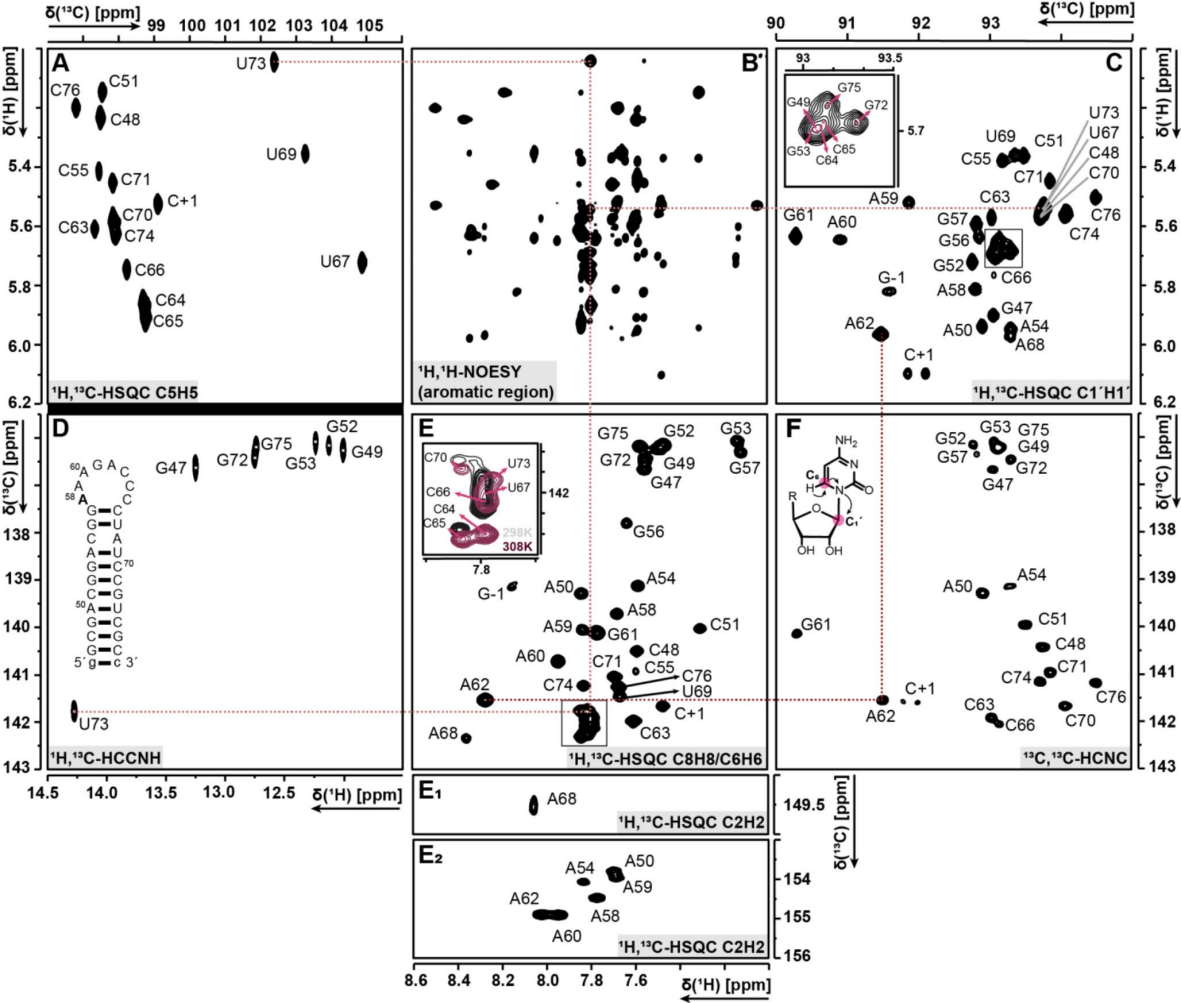
Spectra of the unmethylated RNA (32 nt) construct at pH 6.2 and 298 K **A)**^1^H,^13^C-HSQC (H5-C5 region), **B)**^1^H,^1^H-NOESY (H1’/H5-H2/H6/H8 region), **C)**^1^H,^13^C-HSQC (H1´-C1´ region), **D)**^1^H,^13^C-HCCNH (C6/C8-H1/H3 region), **E)**^1^H,^13^C-HSQC (C6/C8-H6/H8 region), **E**_**1**_**/E**_**2**_**)**^1^H,^13^C-HSQC (C2-H2 region), **F)**^13^C,^13^C-HCNC (C6/C8-C1´region). The arrangement of the spectra is chosen in such a way as to facilitate the visual recognition of the resonance correlations. As examples, A62 and U73 are highlighted, and their corresponding peak assignment is visualized by dotted lines

For the aromatic and ribose resonance assignment, uniformly ^13^C, ^15^N-labelled samples of the 32 nucleotides long unmethylated construct in NMR-buffer (pH 6.2), either in 90% H_2_O/10% D_2_O or in 100% D_2_O, were used. All 32 signals of the aromatic C6-H6/C8-H8 resonances were detected in the ^1^H,^13^C-HSQC. Using a ^1^H,^13^C-HCCNH (Fig. 3D) for uridine H3-C6 and guanosine H1-C8 correlations, C6-H6 and C8-H8 resonances were assigned for the base paired nucleotides in the aromatic ^1^H,^13^C-HSQC (Fig. 3E). For cytidine and uridine, ^1^H,^1^H-TOCSY were used for the selective correlation between H5-H6 resonances leading to the corresponding carbon resonance assignments of C5 (Fig. 3A) and C6 (Fig. 3E) in the ^1^H,^13^C-HSQC.

The assignment of the remaining cytidine and guanosine aromatic C6-H6/C8-H8 signals was achieved using 3D-NOESY-HSQC and ^1^H,^1^H-NOESY spectra for sequential assignment. The latter experiments were also used to obtain the C1´-H1´ (Fig. 3D) resonances, supported by 3D-HCN, 3D-CNC (Fig. 3F) spectra. All C1´-H1´ resonances were successfully assigned, with the exception of C65, due to strong overlap of signals in both ^1^H,^1^H-NOESY and ^1^H,^13^C-HSQC (C1´-H1´) spectra. The remaining ribose resonances (C2´-H2´, C3´-H3´, C4´-H4´, C5´-H5´/-H5´´) were assigned in the ^1^H,^13^C-HSQC (all CH) with the assistance of four 3D-HCCH-TOCSY spectra with two different mixing times and detection on either ^13^C or ^1^H in F1 (see Table 1). Additionally, ^1^H,^15^N-HSQC was used for the assignment of the resonances for N1-/N3-H2 and N7-/N9-H8 of adenosine and guanosine. The assignment of the amino signals was limited to the canonical cytidines.

### The AC-rich octa-loop

**Fig. 4:**
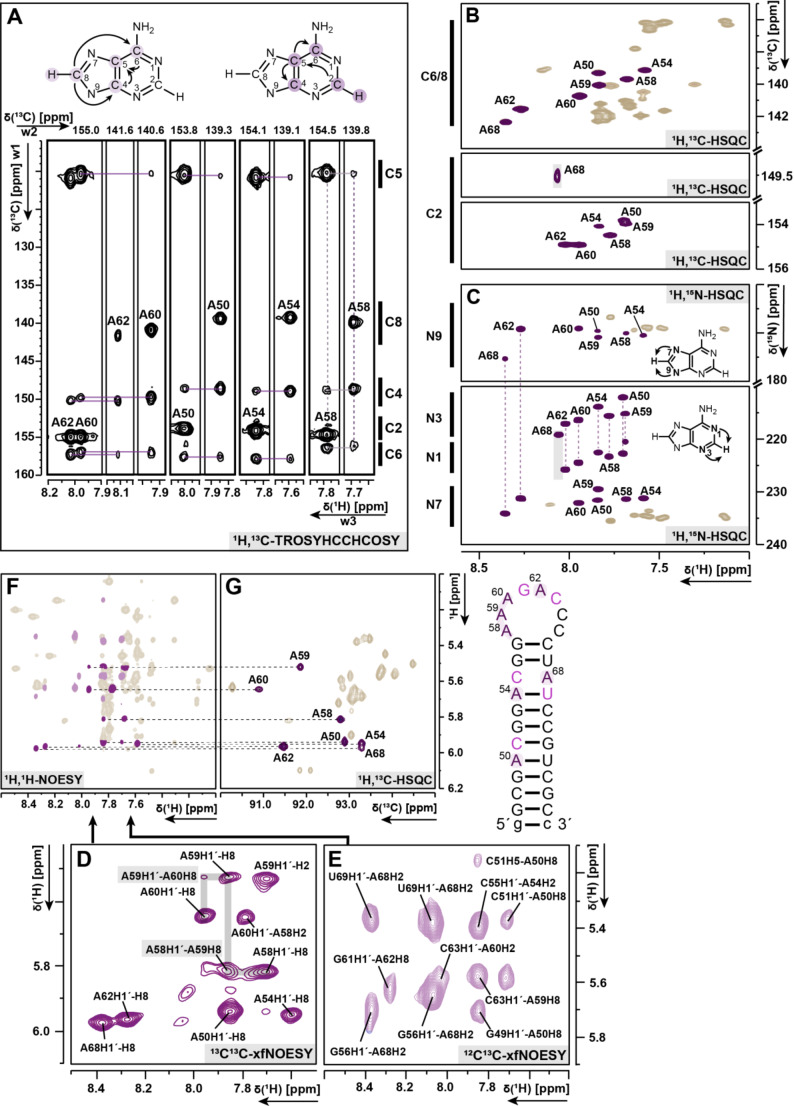
Spectra of the selective ^13^C,^15^N-adenosine unmethylated RNA (32 nt) construct **A**) 3D ^1^H,^13^C-TROSY-HCCH-COSY **B**) ^1^H,^13^C-HSQC (aromatic region) with purple colored adenosine **C**) ^1^H,^15^N-HSQC (N1-/N3-H2 and N7-/N9-H8) with purple colored adenosines ^1^H,^1^H-NOESY with **D**) ^13^C,^13^C-xfilter, **E**) ^12^C,^13^C-xfilter **F**) ^1^H,^1^H-NOESY with D) in purple and F) in light purple marked, all remaining signals are colored in soft beige and **G**) ^1^H,^13^C-HSQC (C1´-H1´ region) with purple colored adenosines

Our resonance assignment implies that the unmethylated construct contains a 5`-AAAGACCC-3`octa-loop, which is rich in adenosines and cytidines. The first adenosine (A58) is dimethylated by Erm proteins in a two-step reaction. This methylation induces antibiotic resistance. Thus, the chemical shift assignment of A58 is of particular interest. To facilitate the assignment of the four loop adenosines, a selective ^13^C, ^15^N- adenosine labeled sample was prepared and measured. All C8-H8 resonances were identified by sequential assignment. For each C8-H8 the corresponding C2-H2 resonance was assigned by using the 3D-TROSY-HCCH-COSY experiment (Fig. 4A) as well as most of the adenosine C4 (86%), C5 (86%) and C6 (71%).

The resonances of the stem-loop cytidines C63 and C64 were obtained by sequential assignment using a ^1^H,^1^H-NOESY, supported by 3D-HCN and ^1^H,^1^H-TOCSY. The H5, H6 resonances of C65 were identified by NOE signals between H5 of C65 to H6 of C64 and H5 of C66 to H6 of C65.

### The stem and the AC mismatch

Interestingly, C2-H2 of A68 (Fig. 4B), located in the mismatch, displays a strong upfield shift in the carbon dimension and is only visible at temperatures above 298 K. This behavior is characteristic for transient protonation at position N1 (Wolter et al. 2017; Toews et al. 2024; Zhu et al. 2024). The filtered NOESY experiments (Fig. 4D, E) with ^13^C,^13^C- and ^12^C,^13^C -filter were used to support the assignment within the loop. ^1^H,^15^N-HSQC (Fig. 4C) of the selectively labelled sample confirmed the correlation signals between N7/9 to H8 and N1/3 to H2 via ^2^J_H, N_ couplings for the adenosines. In the latter experiment A68 did only show a signal for N3-H2, but not for the N1-H2, supporting its assumed transient protonation state.

The C55-A68 mismatch is separated from the loop by only two base pairs, the GC closing base pair and a G56-U67 wobble base pair. The stem contains two canonical AU base pairs, one directly adjacent to the CA mismatch (A54-U69) and A50-U73 in the lower stem. Interestingly, the C6-H6 and C1´-H1´ resonances of U67 and U73 overlap with the cytidine resonances C65 and C64. Therefore, a ^13^C,^15^N-A/U -labelled sample was prepared and used to identify the uridines. Signal overlap of the resonances was greatly reduced. U67 and U73 resonances are very close to each other and difficult to distinguish for the ribose resonances, especially since U67 signals are weaker. However, the ribose resonance assignment for all uridines except for U67 was achieved.

### Comparison of the unmethylated RNA with the m6A methylated RNA

**Fig. 5:**
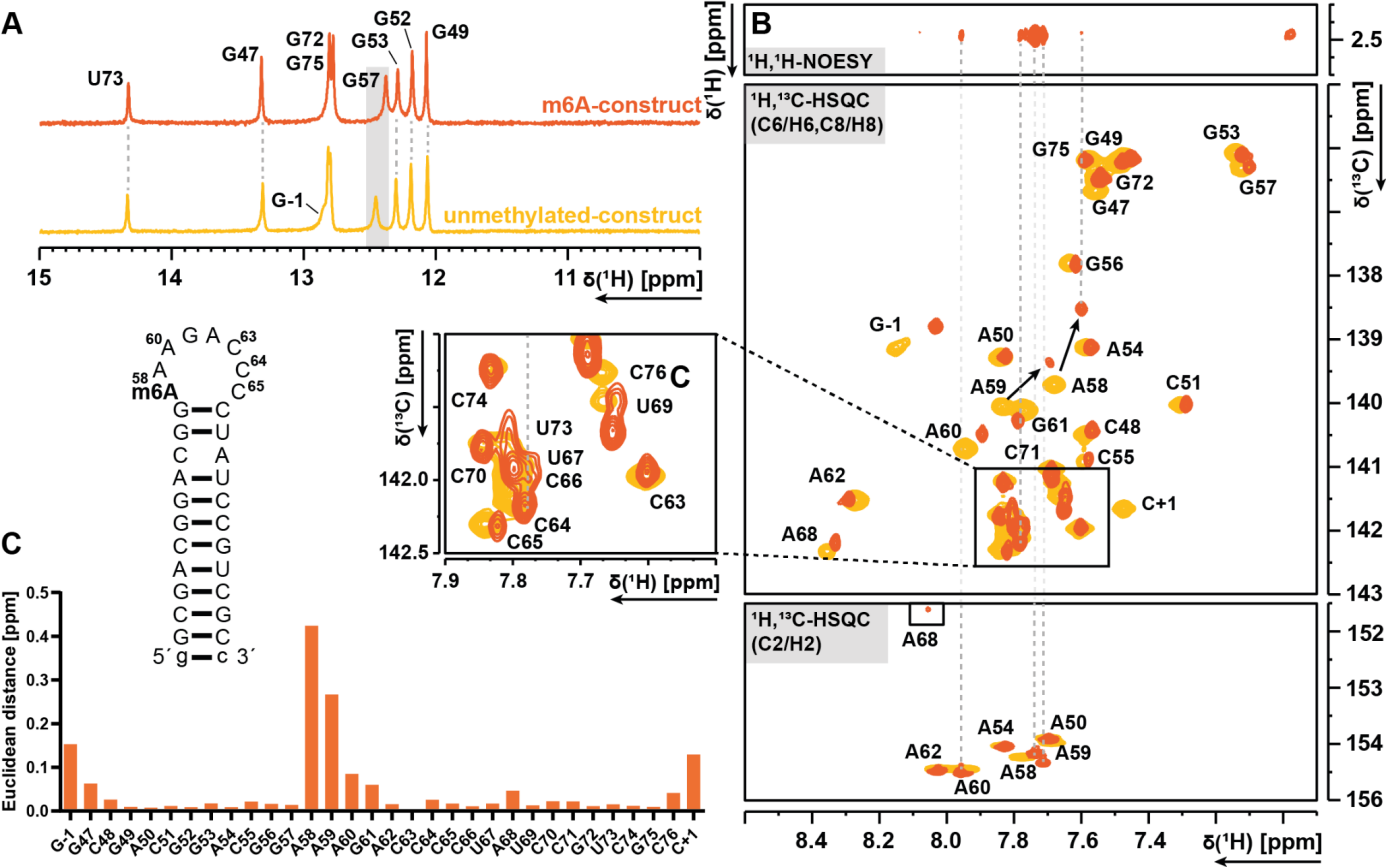
**A**) 1D ^1^H-Spectra overlay showing nearly identical imino proton signals for the unmethylated RNA construct (bottom) and m^6^A-construct (upper). G57 is marked by a grey background, indicating a minor shift. **B**) ^1^H,^1^H-NOESY (methyl region to aromatic region) for the correlations to the H2 and H8 resonances to the ^1^H,^13^C-HSQC (unmethylated RNA (yellow), m^6^A-construct (orange)), **C**) CSP calculations for the aromatic chemical shifts from the methylated m^6^A-construct to the unmethylated construct. All spectra were recorded at 298 K in NMR-buffer in H_2_O/D_2_O 19:1 at pH 6.2

In the first step towards MLS resistance, N6 of the A58 is monomethylated. To capture the changes of chemical shifts caused by methylation, an RNA construct (32 nt) with m^6^A58 synthesized by Dharmacon was used. The ^1^H-1D spectra (Fig. 5A) show a nearly identical imino proton signal pattern compared to the unmethylated RNA. ^1^H,^1^H-NOESY, ^1^H,^1^H-TOCSY, ^1^H,^13^C-sfHMQC and ^1^H,^13^C-HSQC spectra were measured (see Table 2). As the resonances of the m^6^A construct predominantly display minor shifts, most of the assignment could be directly transferred from the unmethylated construct. As the CSPs (see Fig. 5C) of the aromatic region demonstrate, there are only small changes in the chemical shift of almost all stem resonances, including G49 to G57 and U69 to G75, and even the loop closing base pairs. The CSPs of the terminal base pair (g-1/c + 1) are larger due to the difference in the phosphorylation state of c + 1 (the 3´-terminal residue). This difference results from in vitro transcription and HDV cleavage (2’3’-cyclic phosphate) as compared to solid-phase synthesis (3’-OH). These CSPs slightly extend to neighbouring base pairs. The loop nucleotides surrounding A58 demonstrate a divergent pattern, with stronger shifts being observed for the 5’-half of the octaloop, including A58/59/60 and G61. Interestingly, the 3’-half (A62-C65), exhibits no significant effect from the methyl group, similar to the stem. M6A modifications can have diverse effects on RNA structure and stability, depending on their precise structural context (Zhou et al. 2016; Liu et al. 2018, 2021; Jones et al. 2022; Becker et al. 2024). Here, the m6A modification in the loop appears to have a more limited effect. Assignment of the stronger shifting resonances (A58/59/60 and G61) was conducted using the ^1^H,^1^HNOESY. The sequential assignment confirmed the correct transfer of the assignment and proved the prior assignment of the unmethylated construct to be correct. Furthermore, the strongly overlapping signals of the uridines and cytidines at around 142 ppm showed better resolution in the m^6^A58 RNA (see Fig. 5B). Finally, all aromatic C8-H8, C6-H6, C2-H2 as C5-H5 and all observable imino resonances of the m^6^A58 RNA were successfully assigned. Using the ^1^H,^1^H-NOESY and ^1^H,^13^C-HSQC spectra, all C1´-H1´ resonances, except C65 were assigned.

The methyl group of the m^6^A construct resonates at 2.49 ppm. Interestingly, the methyl group at position N6 of A58 exhibits NOE contacts not only to H2 of A58, but also to H2 of A59 and A60. This observation provides a plausible explanation for the resulting strong CSPs for A59 and A60 and the reason why G57 remains unaffected.

### Resonance assignment of the DMA (dimethylated RNA)

The next step was to compare the dimethylated construct to the m^6^A- and the unmethylated construct. As the CSPs of the first methylation step (m^6^A-construct) showed negligible impact on the stem, we decided to subsequently use a truncated stem for the DMA-construct with focus on minimizing the severe overlap.

**Fig. 6:**
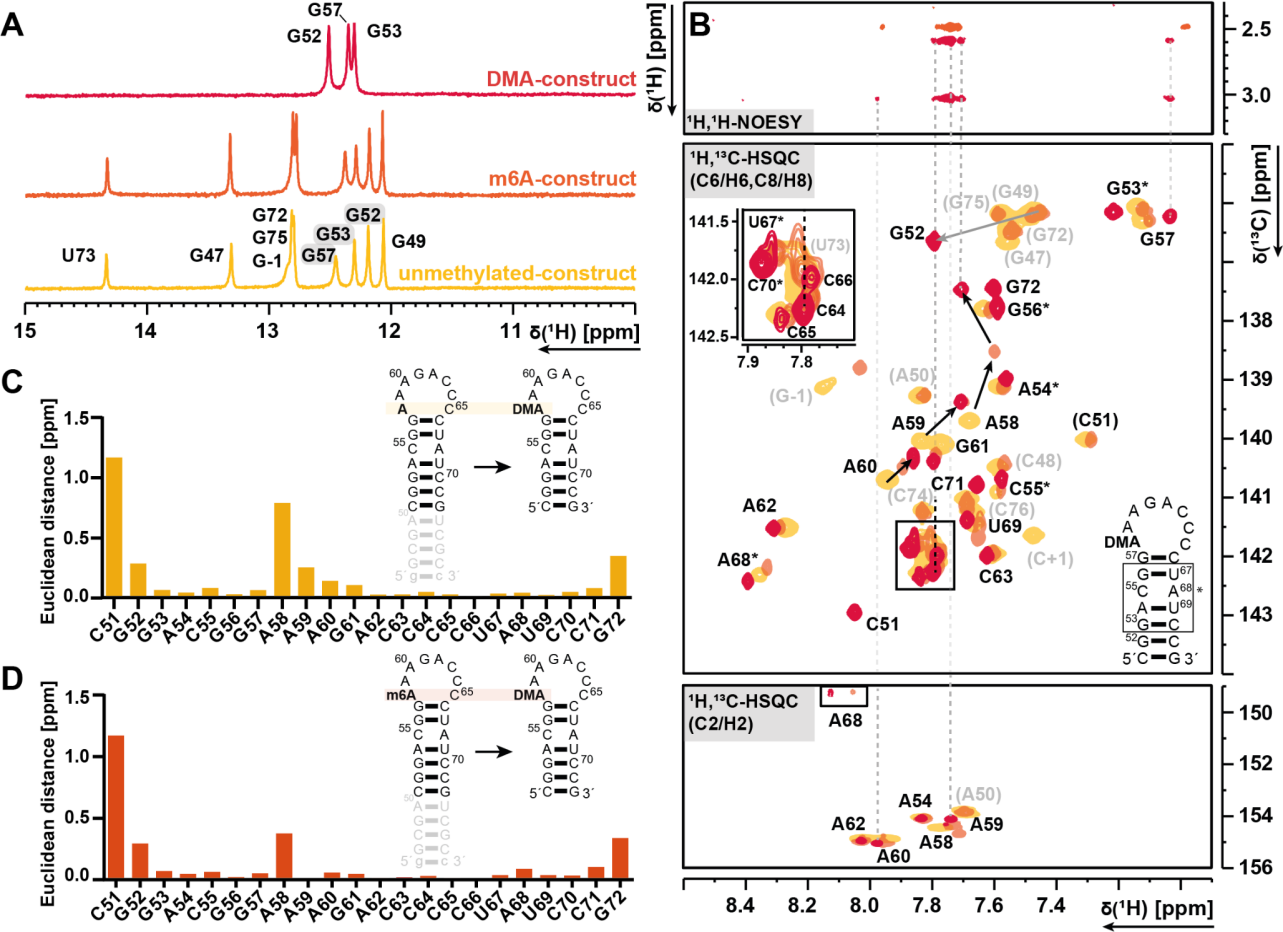
**A**) 1D ^1^H-Spectra overlay of the unmethylated RNA construct (yellow), m^6^A-construct (orange) and DMA-construct (red), **B**) ^1^H,^1^H-NOESY (methyl region to aromatic region) for the correlations to the H2 and H8 resonances to the ^1^H,^13^C-HSQC for all three constructs (unmethylated RNA (yellow), m^6^A-construct (orange) and DMA-construct (red), CSP calculations for **C**) unmethylated-/DMA-construct and **D**) m^6^A-/DMA-construct. All spectra were recorded at 298 K. DMA construct was truncated compared to the unmethylated-/m^6^A construct. Consequently, the corresponding grey colored sequence was not taken into consideration when determing the CSPs. NOE contacts from the methyl groups are demonstrated by dotted lines. Grey numbers belong to the truncated sequence

All imino proton signals expected at 298 K were obtained. While G52 and G53 swapped positions, G57 showed a higher signal intensity than in the unmethylated- and m^6^A-RNA. (Fig. 6A). At 278 K, also the imino proton signals of G72 (g + 1), U69, G56 and U67 were observed (SI Fig. 2). ^1^H,^1^H-NOESY, ^1^H,^1^H-TOCSY, ^1^H,^13^C-sfHMQC and ^1^H,^13^C-HSQC spectra were measured (see Table 2). The assignment was transferred from the m^6^A-construct, and for the stronger signal shifts, the sequential assignment in the ^1^H,^1^H-NOESY was used to obtain both aromatic (C6-H6/C8-H8) and ribose (C1´-H1´) resonances. Consequently, the assignment of all aromatic C8-H8, C6-H6, C2-H2 as C5-H5 of the DMA RNA-construct was successfully achieved. With regard to the ribose CH1´ resonances, the assignment of the C1´-H1´ resonances was successful for all residues except C65 and C66.

The CSPs of the aromatic region (C6-H6/C8-H8) were larger for the terminal base pairs, as expected, due to the truncation of the stem. Figure 6 shows the comparison between both unmethylated and methylated constructs and the dimethylated construct (see Fig. 6C, D). Interestingly, the second methylation has no effect on A59 and a small effect on G57, while the first methylation has a significant effect on A59.

For both methyl groups NOE signals to H2 of A60 and A59 were obtained, as well as weak NOE signals to H8 of C64, A58 and G57 suggesting the positioning of the adenosine into the stem-loop.

## Conclusion

Antibiotic resistance represents a major concern that is growing increasingly more significant. MLS-resistance occurs when the adenosine 2058 (*E. coli* numbering) is dimethylated in position N6 in a two-step reaction with SAM as cofactor. Shorter constructs derived from the relevant sequence have been shown to be methylated by proteins of the Erm family, thus facilitating the investigation of the complex process. Here we report the nearly complete ^1^H, ^13^C, ^15^N chemical resonance assignment of this relevant unmethylated RNA construct by solution NMR. The complete assignment of the imino proton signals allowed us to present the correct secondary structure, which differs from the previously reported one. The correct secondary structure of the 32-nucleotide long unmethylated RNA construct features a pH-sensitive C-A-mismatch replacing the suggested C/AU bulge, resulting in a G56-U67 and G57-C66 base pair instead of two GC base pairs as previously described. This “register shift” of the sequence leads to the formation of an octaloop. Since the Erm-RNA complex is an attractive subject for simulations and 3D structure predictions, this new information should prove beneficial. Furthermore, A68 forming the C-A mismatch was found to be protonated. However, no additional imino signal is observable, even at lower pH values. Remarkably, at a pH of 5.2, the previously absent imino proton signal of U69, and the NOE contact between G56 and U67 are observable. Both base pairs are located directly adjacent to the C55-A68 mismatch.

All imino resonances were assigned for the unmethylated construct, as well as 100% of the aromatic resonances (C6-H6, C8-H8, C5-H5, and C2-H2). A 3D TROSY-HCCH-COSY was used to correlate all C8-H8 resonances with their corresponding C2-H2 and revealed the resonances of the quaternary carbon resonances for C4, C5 and C6. A total of 97% of the C1´-H1´ resonances were assigned, except for C65, which was not unambiguously assignable due to significant signal overlap. 94% of the ribose assignment was completed for C2´-H2, 91% for C3´-H3´, C4´-H4´and C5´-H5´and 53% for the H5´´ resonances. Furthermore, 62% of the G-N1/U-N3 resonances were assigned by ^1^H, ^13^C-HCCNH at 298 K. The assignment of all N1, N3, N7 and N9 resonances of the adenosines and N7 and N9 resonances of the guanosines was achieved by ^1^H, ^15^N-HSQC via ^2^Jcouplings, except for the resonances for N1 of A68 and N9 for g-1. A total of 93% of the N1 resonances for cytidine and uridine were assigned by using 3D HCN.

In addition, a comparison revealed only local differences between the unmethylated RNA construct and the methylated m^6^A-construct. The ^1^H-1D spectra display nearly identical imino proton resonances. The CSPs (Fig. 5C) of the aromatic resonances indicate that methylation exerts a significant influence on the 5’-region of the loop, while stem resonances exhibit only negligible CSPs. These minor changes allowed transfer of the NMR assignment from the unmethylated to the m^6^A construct. At all temperatures measured, the imino proton resonance patterns remain consistent. The truncated DMA-construct provides spectra with less severe overlap in the pyrimidine region. However, most assignments are directly transferrable between the different constructs. The second methylation event has less structural impact on A59, as evidenced by globally small CSPs, while the methyl groups exhibit NOE contact to H2 of A59.

In general, the resonance assignment presented here establishes the foundation for subsequent NMR investigations, particularly with regard to the RNA-protein complex studies. It also serves integrative approaches, which are used with increasing prevalence in structural biology (Schwalbe et al. 2024). We map here the methylation effects with atomic resolution. This work demonstrates the impact of methylation on the Erm target RNA and provides important reporter signals for future work.

## Electronic supplementary material

Below is the link to the electronic supplementary material.

Supplementary Material 1

## Data Availability

The Data Deposition for all three constructs was uploaded to BMRB. Data can be found for the unmethylated construct in BMRB with the code 52938, for the mono-methylated (m6A) construct with the code 52937 and for the dimethylated (DMA) construct with the code 52936.Experimental raw have been deposited under 10.25716/gude.0wv1-r4p4.
