# Supplementary material for: NMR characterisation of the antibiotic resistance-mediating 32mer RNA from the 23S ribosomal RNA
Source: Biomol NMR Assign. 2025 Apr 3;19(1):133–45. doi: 10.1007/s12104-025-10229-2 (PMC12116650; doi:10.1007/s12104-025-10229-2)
Supplement: Supplementary file 1 — Supplementary Material 1 [file 12104_2025_10229_MOESM1_ESM.docx]

**Supplementary information**

for

**NMR characterisation of the antibiotic resistance-mediating 32mer RNA from the 23S ribosomal RNA**

Christina Muhs^1^, Lena Kemper^1^, Christian Richter^1^, Francesca Lavore^2^, Markus Weingarth^2^, Anna Wacker^1^, Harald Schwalbe^1^

^1.^ Center for Biomolecular Magnetic Resonance (BMRZ), Institute for Organic Chemistry and Chemical Biology, Frankfurt am Main, Goethe University, Max-von-Laue-Straße 7, 60438 Frankfurt am Main, Germany

^2.^  NMR Spectroscopy, Department of Chemistry, Utrecht University, Padualaan 8, 3584 CH Utrecht, the Netherlands

Keywords: **m6A, DMA, erythromycin methyl transferase, antibiotic resistance, solution NMR-spectroscopy, 23S ribosomal RNA**


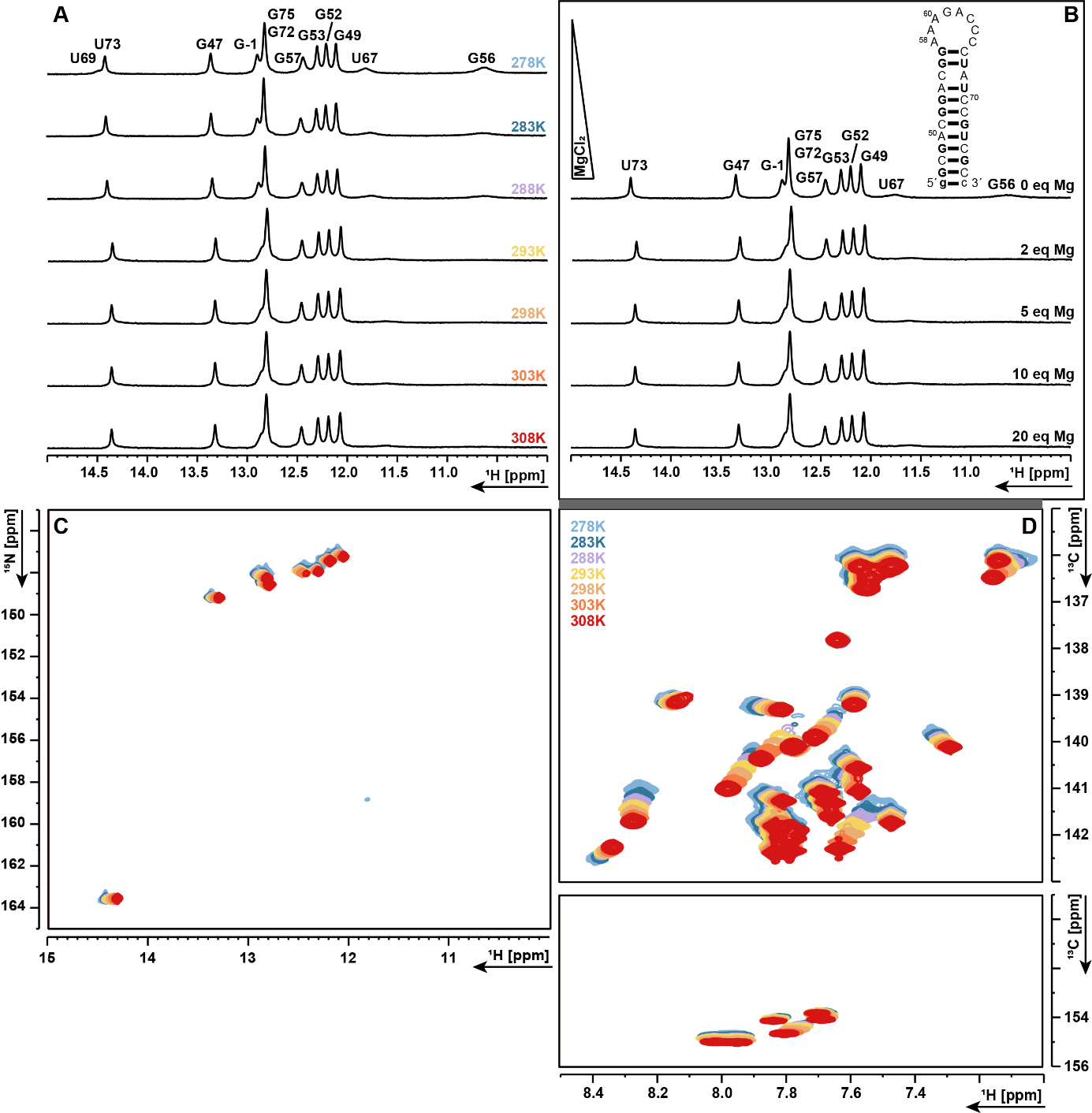


Figure SI 1: A) ^1^H-1D spectra of the unmethylated RNA construct measured within a temperature range of 278 K to 308 K. B) ^1^H-1D spectra were measured at 278 K with varying MgCl_2_ concentrations (0 eq – 20 eq) to an RNA concentration of 185 µM. Temperature series were measured for C) ^1^H,^15^N-best-TROSY D) ^1^H,^13^C-HSQC.


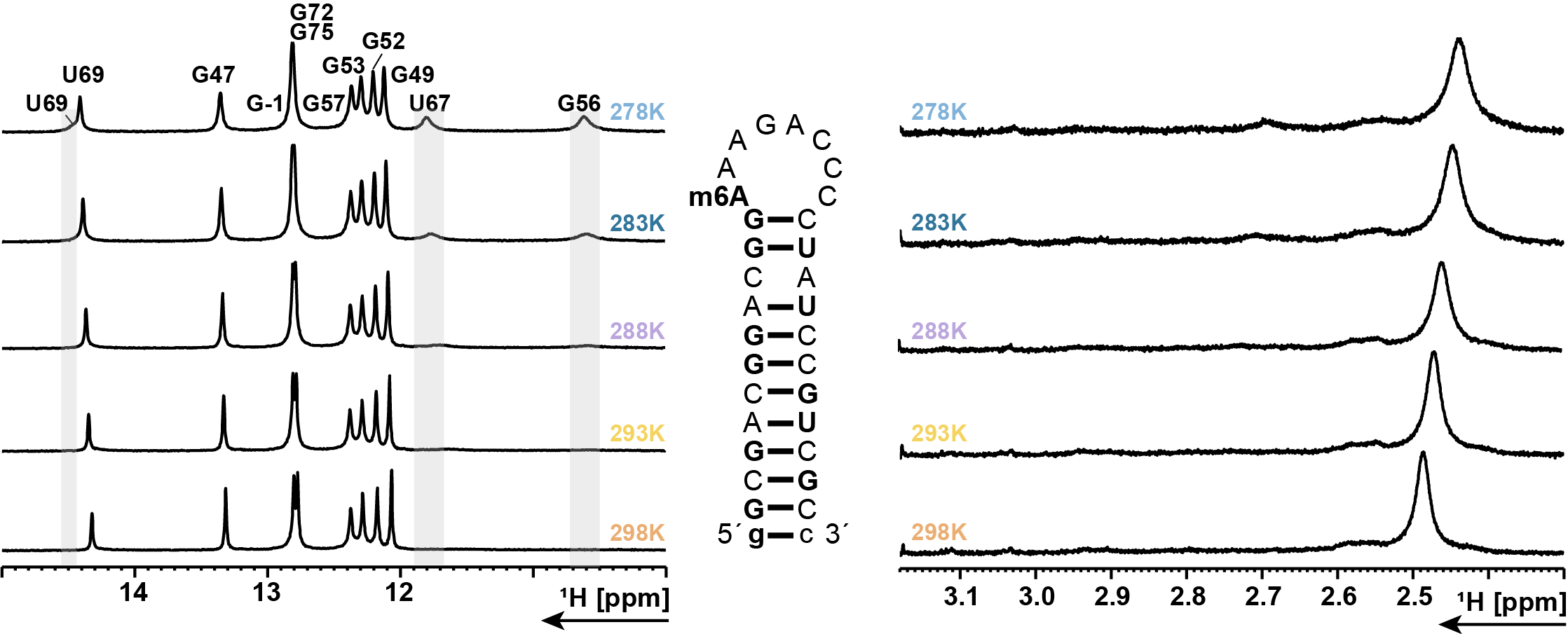


Figure SI 2: ^1^H-1D spectra of the m^6^A-construct measured in a temperature range of 278 K to 298 K. The left side of the figure displays the imino-proton signal region, while the methyl group signal is shown on the right.


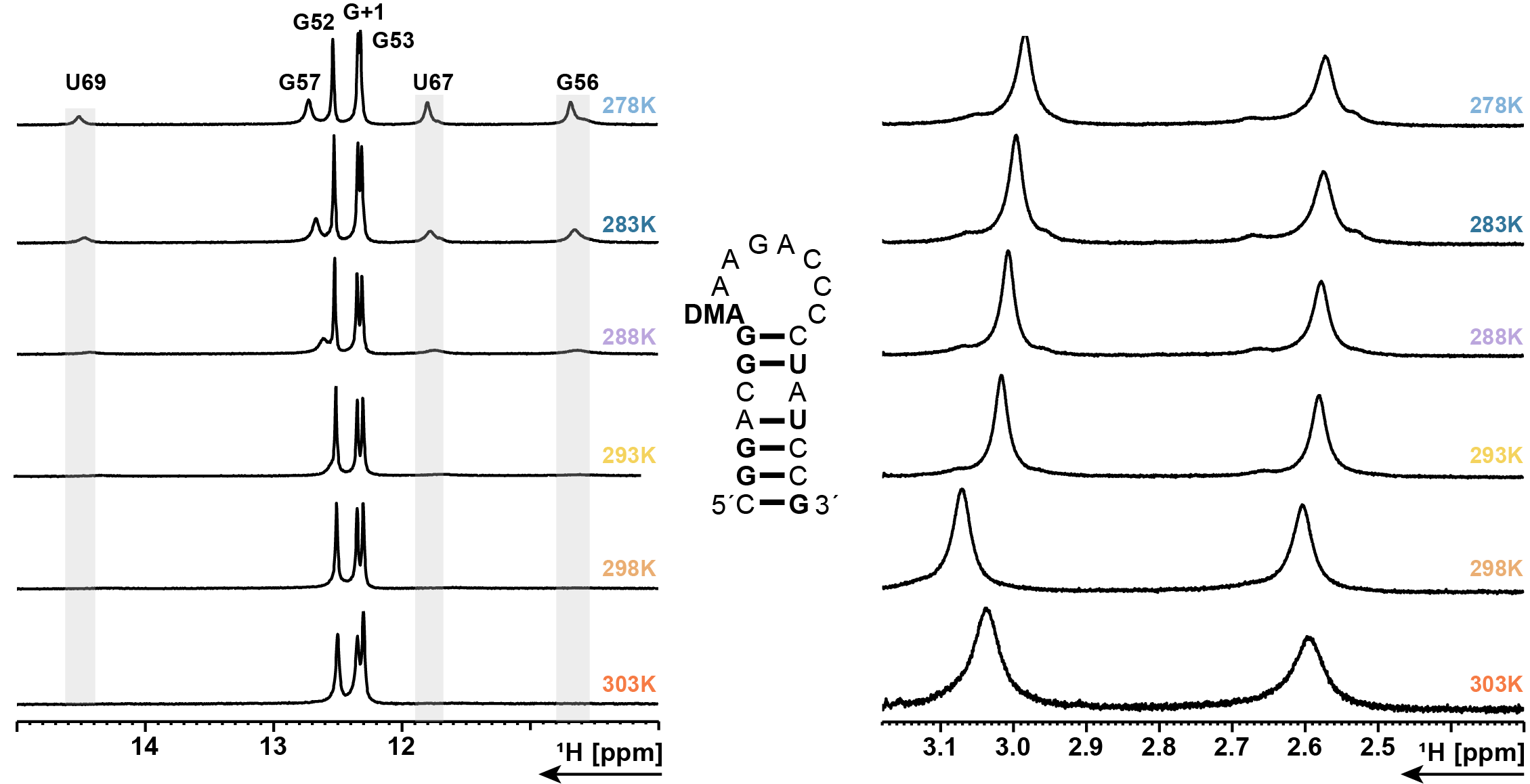


Figure SI 3: ^1^H-1D spectra of the DMA-construct measured within a temperature range of 278 K to 298 K. The left side of the figure displays the imino-proton signal region, while the methyl group signals are shown on the right.


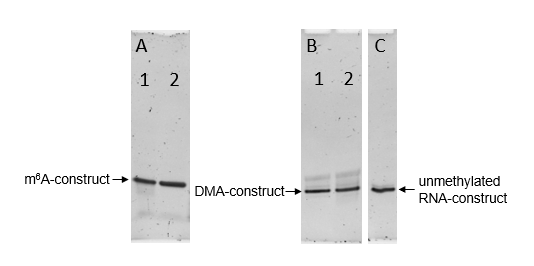


Figure SI 4: Purity of the RNA samples: A) Di-methylated (DMA) – construct before (1) and after (2) HPLC purification. B) Mono-methylated (m^6^A) – construct after (1) and before (2) HPLC. C) unmethylated RNA - construct after HPLC. Conditions for denaturing PAGE: A) 12% PAGE, 30 min @ 240 V; B) 15% PAGE, 35 min @ 260 V; C) 15% PAGE, 35 min @ 260 V.
